# Association between Birth Order and Emergency Room Visits and Acute Hospital Admissions following Pediatric Vaccination: A Self-Controlled Study

**DOI:** 10.1371/journal.pone.0081070

**Published:** 2013-12-04

**Authors:** Steven Hawken, Jeffrey C. Kwong, Shelley L. Deeks, Natasha S. Crowcroft, Robin Ducharme, Douglas G. Manuel, Kumanan Wilson

**Affiliations:** 1 ICES uOttawa, Ottawa, Ontario, Canada; 2 Department of Clinical Epidemiology, Ottawa Hospital Research Institute, Ottawa, Ontario, Canada; 3 Department of Epidemiology and Community Medicine, University of Ottawa, Ottawa, Ontario, Canada; 4 Public Health Ontario, Toronto, Ontario, Canada; 5 Dalla Lana School of Public Health, University of Toronto, Toronto, Ontario, Canada; 6 Department of Family and Community Medicine, University of Toronto, Toronto, Ontario, Canada; 7 Institute for Clinical Evaluative Sciences, Toronto, Ontario, Canada; 8 Laboratory Medicine and Pathobiology, University of Toronto, Toronto, Ontario, Canada; 9 Department of Family Medicine, University of Ottawa, Ottawa, Ontario, Canada; 10 Department of Medicine, Ottawa Hospital Research Institute, University of Ottawa, Ottawa, Ontario, Canada; Weill Cornell Medical College, United States of America

## Abstract

**Objective:**

We investigated the association between a child's birth order and emergency room (ER) visits and hospital admissions following 2-,4-,6- and 12-month pediatric vaccinations.

**Methods:**

We included all children born in Ontario between April 1^st^, 2006 and March 31^st^, 2009 who received a qualifying vaccination. We identified vaccinations, ER visits and admissions using health administrative data housed at the Institute for Clinical Evaluative Sciences. We used the self-controlled case series design to compare the relative incidence (RI) of events among 1^st^-born and later-born children using relative incidence ratios (RIR).

**Results:**

For the 2-month vaccination, the RIR for 1^st^-borns versus later-born children was 1.37 (95% CI: 1.19–1.57), which translates to 112 additional events/100,000 vaccinated. For the 4-month vaccination, the RIR for 1^st^-borns vs. later-borns was 1.70 (95% CI: 1.45–1.99), representing 157 additional events/100,000 vaccinated. At 6 months, the RIR for 1^st^ vs. later-borns was 1.27 (95% CI: 1.09–1.48), or 77 excess events/100,000 vaccinated. At the 12-month vaccination, the RIR was 1.11 (95% CI: 1.02–1.21), or 249 excess events/100,000 vaccinated.

**Conclusions:**

Birth order is associated with increased incidence of ER visits and hospitalizations following vaccination in infancy. 1^st^-born children had significantly higher relative incidence of events compared to later-born children.

## Introduction

There is evidence that familial factors such as the number of siblings and birth order may influence development of allergies, asthma and immunologic sensitization [1]–6. Vaccination coverage and compliance may also be impacted through heightened parental anxiety with respect to their children who have an earlier birth order rank [7]. We postulated that birth order could also influence rates of post-vaccination adverse events based on physiological or non-physiological etiologies.

As part of the publicly funded vaccination schedule in Ontario, Canada, the pentavalent diptheria, tetanus, acellular pertussis, inactivated poliovirus and *Haemophilus influenzae* type b vaccine (DTaP-IPV-Hib) is currently given at 2, 4, and 6 months of age, and the first dose of the measles, mumps and rubella vaccine (MMR) is given at 12 months of age. These vaccines have been broadly used in children, and have been proven safe and effective in preventing disease [8]. Both MMR and DTaP-IPV-Hib can cause mild adverse events, while serious reactions are extremely rare [9]–[11]. With DTaP-IPV-Hib, reactions typically occur in the first 72 hours following vaccination, whereas for MMR, a live attenuated vaccine, reactions typically occur 1–2 weeks post-vaccination [9].

In our previous work using the self-controlled case series (SCCS) study design, we found no significant increase in hospital admissions and emergency room (ER) visits in the first 72 hours after the 2-, 4-, or 6-month DTaP-IPV-Hib vaccinations. We noted however that there was a distinct reduction in rates of ER visits and admissions in the periods immediately preceding and following completed vaccinations, which we attributed to a healthy vaccinee bias [11], [12]. The healthy vaccinee bias arises due to the fact that a child who has recently been ill, is more likely to have vaccination deferred either by parental or health care provider choice. Hence the period immediately preceding and following a completed vaccination tends to be a period of good health, which is reflected by lower health services utilization during this period [11]. This bias could have masked a true increase in events immediately following vaccination if such an increase were present. We also identified a significant increase in incidence of ER visits and/or admissions 4 to 12 days after the 12-month MMR vaccination, as compared to a control period (relative incidence of 1.33 (95% CI: 1.29–1.38)) [10]. This result was consistent with previously published physiological findings [12]. We also identified potential risk factors for increased incidence of adverse events following immunization (AEFIs) [13], [14]. We demonstrated the general utility of health services outcomes for evaluating vaccine safety, while also describing an analytical approach that allowed the identification of immediate post-vaccination increases in adverse events that might otherwise be underestimated or missed entirely due to the healthy vaccinee bias [15].

Additional factors may also potentiate health care utilization after vaccination, particularly if they contribute to heightened parental concern over a child's normal physiologic response to a vaccine. We hypothesized that birth order could be a contributing factor as a result of both physiological and non-physiological etiologies. In this study, we investigated the association between birth order and seeking care for AEFIs.

## Methods

The objective of this study was to determine if the relative incidence of adverse events following 2-, 4-, 6- and 12-month vaccinations, defined as all-cause ER visits and acute hospital admissions, is associated with the vaccinated child's rank in the family birth order.

### Ethics statement

Ethics approval for this study was obtained from the Ottawa Hospital Research Ethics Board (OHREB). This study was performed within ICES' status as a Prescribed Entity under Ontario, Canada's privacy legislation, which allows the anonymized health administrative databases at ICES to be used for healthcare research purposes under strict conditions without express consent.

### Data

All children born in Ontario between April 1^st^ 2006 and March 31^st^ 2009 who were enrolled in the Ontario Health Insurance Plan (OHIP) were eligible for initial study inclusion. Follow-up data was available for most children up to March 31^st^ 2011. Of these children, those who were vaccinated at one or more of the 2-, 4-, 6- and 12-month scheduled visits, and who had complete follow-up data for the risk and control periods were included. For each vaccination, we observed children until the end of the control period (18 days post-vaccination for the 2-, 4-, and 6-month vaccinations and 28 days post-vaccination for the 12-month vaccination). We excluded children who died, or whose follow-up otherwise terminated before the end of the required observation period (e.g. if they moved out of province and became ineligible for OHIP). We ascertained pediatric vaccination using general vaccination billing codes in the OHIP database. To identify the 2-, 4- and 6-month vaccinations, we selected vaccinations occurring on the exact due date (60, 120 and 180 days) and up to two weeks before or up to 1 month after the due date. To identify the 12-month vaccination, we selected vaccinations occurring on the exact due date (365 days of age), as well as vaccinations occurring up to 60 days past the due date [10], [11]. We used the Canadian Institute for Health Information's (CIHI's) Discharge Abstract Database (DAD), which captures all hospital admissions in both tertiary and community hospitals, to ascertain hospital admissions. CIHI's National Ambulatory Care Reporting System (NACRS) was used to identify ER visits. Birth order was determined using the Mom-Baby database at the Institute for Clinical Evaluative Sciences (ICES), which is an ICES-derived database that links mothers and babies based on maternal and infant birth records in the DAD, as well as other available health administrative data. By linking siblings together using the Mom-Baby database, which includes data on mothers and babies born beginning in 1988 (18 years prior to the first baby included in our study), we were able to compare birth dates to determine each child's rank within his/her family's birth order. We excluded children from multiple births (twins for example) from our analysis and those children who could not be linked to their mother to identify siblings. We also excluded babies born prematurely (<37 weeks gestation) and those who were in the lowest decile of birthweight for their gestational age (small for gestational age (SGA10)). The Registered Persons Database at ICES was used to ascertain eligibility for OHIP coverage and date of death, if applicable. All datasets were housed at ICES, where individual-level data was anonymized and linkage between datasets was achieved using encrypted health card numbers as unique identifiers.

### Design and Analysis

This study was conducted using the SCCS design [16], [17] and the Vaccine and Immunization Surveillance in Ontario (VISION) analytic architecture [18]. Because we only included individuals who both had an event of interest and were also vaccinated, this may be more appropriately described as a self-controlled risk interval design [19], though the two designs yield identical results for the analysis approach we used. Based on our previous work described in detail elsewhere [10], [11], we designated the 72 hours following vaccination (day 0 to 2) as the risk (exposed) period and days 9 to 18 as the control (unexposed) period for the 2-, 4- and 6-month vaccinations (DTap-IPV-Hib). The DTap-IPV-Hib vaccine is not a live-virus vaccine, and thus reactions are expected to occur immediately in response to the component antigens of the vaccine [8]. The 12-month vaccine (MMR) is a live attenuated vaccine and hence adverse reactions are expected to occur 1–2 weeks following the vaccination, caused by slow replication of the attenuated virus and reactions may present as a mild measles-like illness [8], [12]. We designated days 4 to 12 as the risk period and days 20 to 28 as the control period following vaccination. Days 4–12 following the 12-month vaccination were identified as the appropriate risk period by testing each day following vaccination individually, and identifying those days where risk was significantly elevated after appropriate adjustment for multiple testing [10]. Control periods were designed to be far enough removed from the index vaccination such that the event rate would have returned to a representative baseline level, while not overlapping with subsequent vaccinations. This was especially important in the case of the closely timed 2-, 4- and 6-month vaccinations [10], [11]. Our composite primary outcome included ER visits and hospital admissions. Where multiple events occurred in a risk or control period (e.g. an ER visit leading to an admission) only the first event was counted. Despite being based on a Poisson model, the SCCS methodology is appropriate for rare non-recurrent events, since the time of first occurrence of a rare potentially recurrent event and the times of occurrence of a rare unique event are indistinguishable in practice. Only children who received a target vaccination and had one or more ER visits or hospitalizations in the observation period contribute to the conditional SCCS analysis [17].

The relative incidence (RI) of the outcome during the exposed period as compared to the unexposed period was calculated using a Poisson regression model which included terms for exposure period and for identifying each individual child, thereby allowing each individual to serve as his/her own control and accounting for intra-individual correlation. To compare the RI of our primary endpoint among children with differing birth order rankings, we calculated the relative incidence ratios (RIRs) for each successive birth order ranking (1^st^-born, 2^nd^-born, 3^rd^-born and 4^th^- or later-born) compared to the chosen reference group. Similarly, we compared all children who were 2^nd^-born or later (later-born) to 1^st^-born children. The RIRs are calculated by including interaction terms for (risk interval) x (birth order category) in the conditional Poisson regression model. The parameter estimates for the interaction terms are exponentiated to yield the RIRs. A likelihood ratio test was used to assess statistical significance of the interaction terms (and hence the RIRs) in the fitted regression model [17].

The SCCS model implicitly controls for all fixed individual factors in estimating RI related to the vaccination exposure. When comparing risks across different strata (such as birth order) using RIRs, the potential for differential distributions of important covariates across the strata exists. The calculated RIRs across strata are not implicitly controlled for by the SCCS model in the same way as the RIs. In order to assess the impact of potential confounders/effect modifiers such as family size, maternal age, birthweight and gestational age, we: 1) stratified by these additional factors and compared the RIRs for birth order among strata; and 2) included the additional factors in the SCCS model as covariates to determine whether the observed RIRs were robust to adjustment for the additional factors.

Acuity of ER visits was measured using the Canadian Triage and Acuity Score (CTAS) recorded in the NACRS database. CTAS ratings range from 1 to 5, with 1 representing a severe condition requiring resuscitation and 5 representing a less severe condition requiring non-urgent care [20]. We compared baseline characteristics of children in subgroups of birth order using chi-square tests for categorical variables, t-tests for normally distributed continuous covariates, and the Wilcoxon signed rank test where non-parametric tests were appropriate (e.g. for CTAS scores). All p-values were 2-sided, and all analyses were conducted using SAS version 9.2 (SAS Institute, Cary, NC).

## Results

### Baseline Characteristics

**Figure 1:**
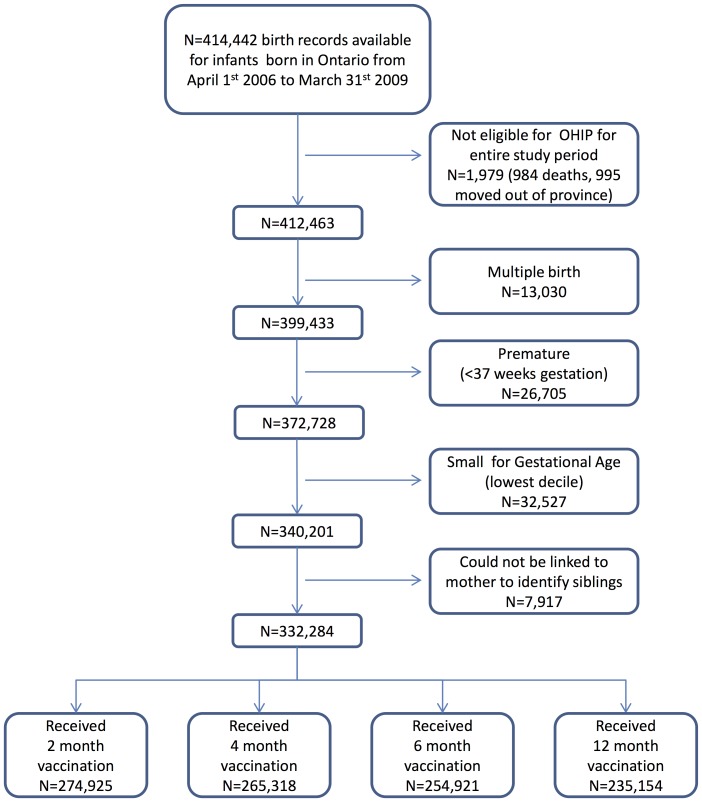
Derivation of Study Cohort.

Of the infants born between April 1^st^ 2006 and March 31^st^ 2009, 274,925 met our study inclusion criteria and received the 2-month vaccination. For the 4-and 6-month vaccination analyses, 265,318 and 254,921 children respectively were eligible and received the target vaccinations. For the 12-month vaccination analysis, 235,154 eligible vaccinated children were included. Figure 1 provides details of the study cohort derivation and the impact of each exclusion criterion on sample size.

Events in the risk periods following the 2-, 4-, 6- and 12-month vaccinations were overwhelmingly (>90%) comprised of ER visits. Both the mean CTAS scores, as well as the proportion of visits that were urgent, emergent or requiring resuscitation (CTAS scores of 1, 2 or 3) vs. non-urgent (CTAS scores of 4 or 5) did not differ between 1^st^-borns and later-borns in any of the risk periods following the 2-, 4-, 6- and 12-month vaccinations (Table 1). Mothers of later-born children tended to be slightly older than mothers of 1^st^-borns. For children vaccinated at 2 months, mothers of later-born children were on average 29.8 years old versus 26.6 years old for mothers of 1^st^-born children (p<0.0001). We observed a small but statistically significant difference in gestational age among 1^st^-borns compared to later-borns (p = 0.0002), as well as a modest trend towards 1^st^-born children having lower birth weight, which did not reach nominal statistical significance (p = 0.11).

**Table 1:** Demographic Characteristics for 1^st^-born children versus later-born children who had an ER visit or admission in the first 72 hours following the 2-month vaccination.

	1st-born Children N = 574	Later-born Children N = 418	p-value for difference
	Mean (SD)	Mean (SD)	t-test p-value
Maternal Age (years)	26.6 (6.0)	29.8 (5.3)	<0.0001
Birth Weight (g)	3486.8 (396.5)	3529.9 (455.4)	0.1074
Gestational Age (Wks)	39.3 (1.23)	39.02 (1.15)	0.0002
	N (%)	N (%)	Chi-square test p-value
Low Income (1^st^ or second neighborhood income quintile)	270 (47.0%)	204 (48.8%)	0.5550
Proportion of events that are ER visits	546 (95.1%)	391 (93.5%)	0.3187
CTAS 1,2 or 3^1^ (Denominator is ER visits)	403 (73.8%)	281 (71.9%)	0.5190

1 Canadian Triage Acuity Score (CTAS): 1 = Resuscitation, 2 = Emergent, 3 = Urgent, 4 = Less Urgent (Semi urgent), 5 = Non Urgent.

### Primary Analysis

**Figure 2:**
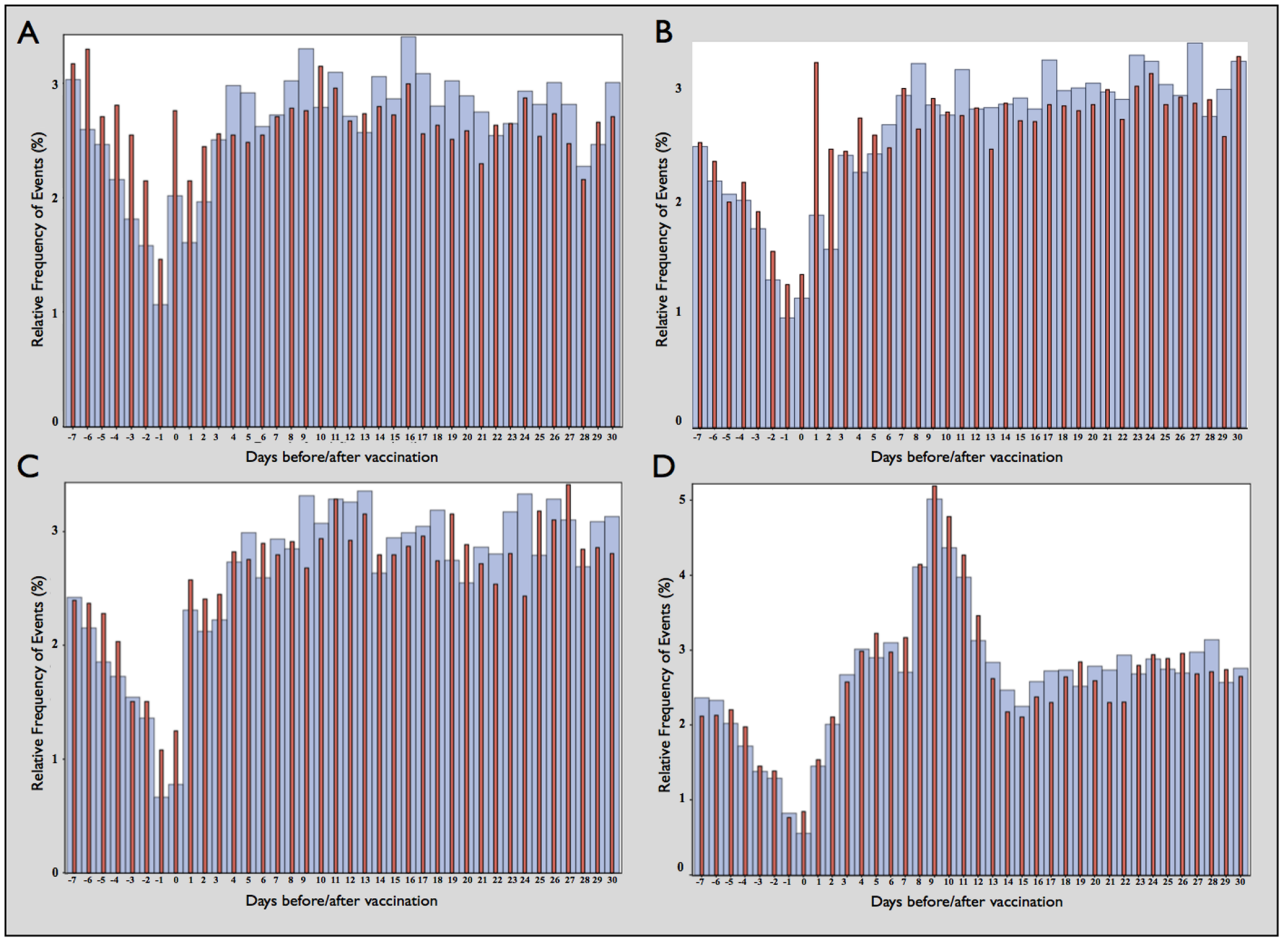
Relative frequency (%) of ER visits and admissions from −7 to +30 days relative to date of vaccination (day 0). A) 2 month vaccination; B) 4 month vaccination; C) 6 month vaccination; D) 12 month vaccination. 1^st^-born: Narrow red bars. Later-born: Wide blue bars.

Figure 2 shows the relative frequency of events on each day relative to the vaccination from 7 days before to 30 days after the date of vaccination (vaccination date = day 0) for each of the 2-, 4-, 6-, and 12-month vaccinations. The frequencies are shown for 1^st^-born children and later-born children separately, showing the different distributions of frequency and timing of events relative to the date of vaccination.

The relative incidences of events in 1^st^-borns versus later-borns and versus 2^nd^-, 3^rd^- and 4^th^- or later-borns for children who received a target vaccination, and who also experienced 1 or more ER visits or admissions are presented in Table 2. In the vast majority of cases, events observed in either the risk or control periods represent distinct individuals, however a small number of children experienced an event in both the risk and control intervals (63 children at the 2-month vaccination out of 4681 total events). For the 2-month vaccination, the overall RI (95% CI) in the risk versus control period was 0.81 (0.75–0.87). For 1^st^-born children, the RI was 0.93 (0.85–1.02) and for 2^nd^- or later-born children it was 0.68 (0.61–0.76). The RIR for 1^st^-borns versus 2^nd^- or later-born children was 1.37 (1.19–1.57, p<0.0001). This translates to 112 additional ER visits or admissions for every 100,000 vaccinated 1^st^-born children compared to children of later birth order, or 1 additional event for every 895 vaccinated 1^st^-born children.

**Table 2:** Adverse Events Following the 2-,4-,6- and 12-Month Vaccination.

Birth order	Vaccinated Children	Events During Risk Period (Days 0–2)*	Events During Control Period (Days 9–18)*	Relative Incidence (95% CI)	Relative Incidence Ratio (95% CI)	RIR p-value∧
**2-month vaccination**						
Overall	274925	992	3689	0.81 (0.75–0.87)	NA	
4^th^ or higher	9513	38	147	0.78 (0.54–1.11)	1 (Ref)	
3	29945	97	403	0.72 (0.58–0.90)	0.93 (0.61–1.42)	
2	98031	283	1290	0.66 (0.58–0.75)	0.85 (0.58–1.24)	
1	137436	574	1849	0.93 (0.85–1.02)	1.20 (0.83–1.74)	0.0002
2^nd^ or higher	137489	418	1840	0.68 (0.61–0.76)	1 (Ref)	
1	137436	574	1849	0.93 (0.85–1.02)	1.37 (1.19–1.57)	<0.0001
**4-month vaccination**						
Overall	265318	794	3284	0.73 (0.67–0.78)	NA	
4^th^ or higher	8718	27	136	0.60 (0.39–0.90)	1 (ref)	
3	28191	73	394	0.56 (0.43–0.71)	0.93(0.58–1.51)	
2	94205	210	1183	0.53 (0.46–0.62)	0.89 (0.58–1.39)	
1	134204	484	1571	0.92 (0.83–1.02)	1.55 (1.01–2.37)	<0.0001
2^nd^or higher	131114	310	1713	0.54 (0.48–0.61)	1 (Ref)	
1	134204	484	1571	0.92 (0.83–1.02)	1.70 (1.45–1.99)	<0.0001
**6-month vaccination**						
Overall	254921	829	3682	0.68(0.63–0.73)	NA	
4^th^ or higher	8046	27	129	0.63(0.41–0.95)	1 (Ref)	
3	26582	80	399	0.60 (0.47–0.76)	0.96 (0.59–1.55)	
2	90100	251	1280	0.59 (0.51–0.67)	0.94 (0.61–1.45)	
1	130193	471	1874	0.75 (0.68–0.83)	1.20 (0.78–1.84)	0.0227
2^nd^ or higher	124728	358	1808	0.59 (0.53–0.67)	1 (Ref)	
1	130193	471	1874	0.75 (0.68–0.83)	1.27 (1.09–1.48)	0.0021
**12-month vaccination**						
Overall	235,154	5595	4158	1.35 (1.29–1.40)	NA	
4^th^or higher	7067	120	98	1.22 (0.94–1.60)	1 (Ref)	
3	24064	474	352	1.35 (1.17–1.55)	1.10 (0.81–1.49)	
2	83021	1744	1396	1.25 (1.17–1.34)	1.02 (0.77–1.35)	
1	121002	3257	2313	1.41 (1.34–1.49)	1.15 (0.88–1.51)	0.0585
2^nd^ or higher	114152	2338	1845	1.27 (1.19–1.35)	1 (Ref)	
1	121002	3257	2313	1.41 (1.34–1.49)	1.11 (1.02–1.21)	0.0108

* Events in risk and control periods overwhelmingly represent distinct individuals, however a small number of children experienced an event in both the risk and control intervals (for example: 63 children had events in both the risk and control periods following the 2 month vaccination out of 4681 total events).

∧p-value for test of differences among levels of birth order categories.

For the 4-month vaccination, the RIR for 1^st^- versus later-borns was 1.70 (1.45–1.99, p<0.0001), translating to 157 additional events for every 100,000 vaccinated 1^st^-borns compared to later-borns, or one additional event for every 636 1^st^-born children vaccinated.

For the 6-month vaccination the RIR for 1^st^-borns compared to later-borns was 1.27 (1.09–1.48, p = 0.0021), translating to 77 additional events for every 100,000 vaccinated 1^st^-borns compared to later-borns, or one additional event for every 1298 1^st^-born children vaccinated.

At the 12-month vaccination, the RIR for 1^st^ versus later-borns was 1.11 (1.02–1.21, p = 0.0108), which translates to 249 excess events for every 100,000 vaccinated 1^st^-borns compared to later-borns, or one excess event for every 401 1^st^-born children vaccinated.

### Secondary and Sensitivity Analyses

We repeated the primary analysis with ER visits alone and acute hospital admissions alone. The results for ER visits were nearly identical to the results for combined events, and the findings for admissions alone showed similar patterns of findings to the combined results, but the precision was much lower given the much smaller number of events.

When we divided 1^st^-borns into 2 groups: 1) those without siblings (only-children as of March 31^st^ 2011), and 2) those with 1 or more younger siblings, the increased RI in 1^st^-borns as compared to later-borns was similar in both groups. For the 2-month vaccination, the RIR comparing 1^st^-borns with siblings to later-borns was 1.46 (1.22–1.74) and that comparing1^st^-borns without siblings to later-borns was 1.30 (1.11-1.53) (Table 3). At the 4-month vaccination, the RIRs were 1.68 (1.37–2.05) and 1.72 (1.44–2.06), at 6 months the RIRs were 1.34 (1.11–1.62) and 1.22 (1.02–1.45) and at 12 months the RIRs were 1.19 (1.08–1.32) and 1.05 (0.96–1.16), respectively (Table 3). In models adjusting for family size (number of siblings as of March 31^st^ 2011), and maternal age (on the birthday of the respective child), the effect of birth order remained robust (Table 4). These findings were also supported by a subgroup sensitivity analysis of families with exactly 3 children and another stratified by categories of maternal age (Supporting Information S1).

**Table 3:** Analyses of 1^st^-borns with and without other siblings (as of March 31^st^ 2011).

Birth order	Events During Risk Period (Days 0–2)	Events During Control Period (Days 9–18)	Relative Incidence (95% CI)	Relative Incidence Ratio (95% CI)	RIR p-value
**2-month Vaccination**					
2^nd^ or higher	418	1840	0.68 (0.61–0.76)	1 (Ref)	
1^st^ with siblings	248	748	0.99 (0.86–1.15)	1.46 (1.22–1.74)	<0.0001*
1^st^ with no siblings#	326	1101	0.89 (0.79–1.01)	1.30 (1.11–1.53)	<0.0015∧
**4-month vaccination**					
2^nd^ or higher	310	1713	0.54 (0.48–0.61)	1 (Ref)	
1^st^ with siblings	198	652	0.91 (0.78–1.07)	1.68 (1.37–2.05)	<0.0001*
1^st^ with no siblings#	286	919	0.93 (0.82–1.07)	1.72 (1.44–2.06)	<0.0001∧
**6-month vaccination**					
2^nd^ or higher	358	1808	0.59 (0.53–0.67)	1 (Ref)	
1^st^ with siblings	213	792	0.80 (0.68–0.93)	1.34 (1.11–1.62)	<0.0026*
1^st^ with no siblings#	271	1082	0.72 (0.63–0.83)	1.22 (1.02–1.45)	<0.0283∧
**12-month vaccination**					
2^nd^ or higher	2338	1845	1.27 (1.19–1.35)	1 (Ref)	
1^st^ with siblings	1450	960	1.51 (1.39–1.64)	1.19 (1.08–1.32)	<0.0007*
1^st^ with no siblings#	1807	1353	1.34 (1.24–1.43)	1.05 (0.96–1.16)	0.2694∧

* p-value for comparison of 1^st^-borns with siblings vs. 2^nd^ or later-borns.

∧p-value for comparison of 1^st^-borns with no siblings vs. 2^nd^ or later-borns.

# As of March 31^st^ 2011.

**Table 4:** Analyses of 1^st^ versus later-borns with Adjustment for Mother's age and number of siblings.

	Adjusted for maternal age at child's birth		Adjusted for number of siblings (as of March 31^st^ 2011)	
Birth order	Relative Incidence Ratio (95% CI)	RIR p-value	Relative Incidence Ratio (95% CI)	RIR p-value
**2-month Vaccination**				
2^nd^ or higher	1 (Ref)		1 (Ref)	
1^st^	1.33 (1.15–1.54)	<0.0001	1.53 (1.26–1.86)	<0.0001
**4-month vaccination**				
2^nd^ or higher	1 (Ref)		1 (Ref)	
1^st^	1.67 (1.42–1.97)	<0.0001	1.71 (1.38–2.13)	<0.0001
**6-month vaccination**				
2^nd^ or higher	1 (Ref)		1 (Ref)	
1^st^	1.25 (1.06–1.46)	0.0063	1.36 (1.11–1.67)	0.0032
**12-month vaccination**				
2^nd^ or higher	1 (Ref)		1 (Ref)	
1^st^	1.09 (1.01–1.19)	0.0370	1.20 (1.07–1.33)	0.0013

## Discussion

Our analysis has shown that, as compared to 2^nd^- or later-born children, 1^st^-born children have a higher relative incidence of AEFIs for which care is sought, defined as emergency room visits and hospital admissions during an at-risk period (as compared to a control period). This increased relative incidence was present at all of the vaccination times examined, but was most apparent at the 2- and 4-month vaccinations, and could not be explained by differences in maternal age, family size, birth weight, or gestational age. Our conclusions were unchanged when we repeated our primary analysis with ER visits and admissions separately. Overall, our results were consistent with our previous findings. The relative incidences of less than one for post-vaccination ER visits and admissions immediately following the 2-, 4- and 6-month vaccinations are attributable to the healthy vaccinee effect. The relative incidence of greater than one from 4 to 12 days following the 12-month vaccination is consistent with the biological mechanism of action of the MMR vaccine [10], [11]. Further stratifying by birth order, we have demonstrated the utility of RIRs to detect differential effects in subgroups in situations where an overall effect in the risk period may be partially or completely masked by the healthy vaccinee effect, and also where the post-vaccination risk period is farther removed from vaccination and thus less likely to be affected by the healthy vaccinee effect (as is the case for the 12-month MMR vaccination).

Other studies have reported on the impact of birth order on vaccination coverage and compliance [7], however, to the best of our knowledge, ours is the first study to examine the association between the relative incidence of AEFIs, either in terms of overall health services utilization or specific types of AEFIs, and birth order of the vaccinated child.

We hypothesize that a portion of the observed excess post-vaccination ER visits and admissions may be due to heightened parental concern over the normal physiological response produced by vaccines in the process of conferring immunity. We would expect heightened parental concern to be particularly characteristic of first-time parents. The higher relative incidence of events we observed for 1^st^-born as compared to later-born children, and the fact that this difference was most pronounced at the first two infant vaccination visits (i.e. at 2 and 4 months of age) suggests that the observed differences may be at least partly driven by either elevated concern in first-time parents, or an evolving decision-making process based on experiences with previous children. Based on our results, if the relative incidence of events following vaccination in 1^st^-borns were reduced to that observed in 2^nd^- or later-born children, this would result in an avoidance of approximately 766 ER visits and admissions annually, for children receiving the full course of 2-, 4-, 6- and 12-month vaccinations, assuming the current birth rate of about 140,000 births per year in Ontario, Canada.

The concepts of parental concern and birth order in relation to vaccine administration in children have previously been studied. Authors of a pilot study conducted in parents of 2-month old infants and toddlers entering clinical trials either of a DaPT or Meningococcal-C vaccine, respectively, reported that among other important predictors, earlier birth order rank was associated with higher parental anxiety scores [21]. Another study reported that for cases of illness, 1^st^-born boys were most frequently taken to the ER, and later-born girls were taken the least often [22]. Verbal reports from parents confirmed that inexperienced first-time parents were more anxious about their children's health than parents of later-born children [22]. In a study examining the relationship between parental anxiety and contact with an outpatient well-child clinic after a child's first DPT vaccination at 2 months of age, Hatcher et al. [23] found that mothers who reported anxiety, as well as those who had infant girls, were more likely to contact the clinic within 72 hours of the DPT vaccination. Physicians are encouraged to inform their patients of expected adverse reactions following vaccination to potentially alleviate parental anxiety [24], [25]. This may be particularly important for first-time parents.

Previous studies have suggested there may be a protective effect from larger numbers of siblings and/or later birth order with respect to development of allergies and asthma, and general immunologic sensitization. Proposed mechanisms for this have included maternal factors, intrauterine environment, placental factors, and the “hygiene hypothesis”, which postulates that a lack of exposure to infectious agents, microbial flora and parasites in early childhood increases susceptibility to allergic disorders by inhibiting the development of the immune system [1]-[6]. We did not observe a dose-response relationship between number of siblings and relative incidence of AEFIs, rather we observed an apparent threshold effect where 1^st^-born children were at increased risk compared to any subsequent child in the birth order and being 3^rd^-born, for example, did not appear to be more protective than being 2^nd^-born. With respect to maternal factors, our study suggested that the birth order effect we observed was independent of maternal age, but we could not rule out the potential effect of other unmeasured maternal factors. Further study is warranted to determine the underlying basis of the differences among children of differing birth order and specifically, whether it is due purely to parental behavior, or whether there is a physiological component to our findings.

This study had a number of strengths and limitations. Firstly, one of our study's strengths was the large sample size, including the vast majority of children born in Ontario, Canada during a two and a half-year span from April 2006 to December 2009. Secondly, by employing the ICES Mom-Baby database, we were able to establish birth order for the vast majority of the children included in the study without requiring alternate data sources or primary data collection. Our use of aggregate health services data as an outcome could be considered as a strength and a weakness. Although much less specific than using targeted health outcomes, this approach is extremely sensitive for capturing ER visits and admissions. However, adverse events that were not severe enough to result in an ER visit would be missed. Furthermore, we used general OHIP vaccination billing codes, which in most cases reflect the targeted vaccines at each time point, but could conceivably have represented a different vaccine than intended in a small number of cases. One other potential limitation was that the Mom-Baby database would not capture births that occurred outside of Ontario, and hence if older siblings were born out of province, a small percentage of babies may be misclassified with respect to birth order. Although we were able to explore the impact of adjustment for a limited number of potential confounders on our RIR estimates, other factors that we were unable to measure (e.g. maternal level of education and previous history of vaccine adverse events) might be important. Our study has demonstrated that birth order is predictive of AEFIs as measured by ER visits and hospitalizations during an at-risk period. Future studies should aim to determine if a proportion of the health service utilization following vaccination could be mitigated through better communication of expected reactions, and whether there is a physiological basis to the phenomenon we observed.

## Supporting Information

Information S1 **Supporting information tables.** Table S1: Analyses of 1st versus later-borns in families with exactly 3 children (as of March 31^st^ 2011); Table S2: Analyses of 1st versus later-borns Stratified by Mother's age.
(DOCX)
