# Supplementary material for: Association between Birth Order and Emergency Room Visits and Acute Hospital Admissions following Pediatric Vaccination: A Self-Controlled Study
Source: PLoS One. 2013 Dec 4;8(12):e81070. doi: 10.1371/journal.pone.0081070 (PMC3852020; doi:10.1371/journal.pone.0081070)
Supplement: Information S1 — Supporting information tables. Table S1: Analyses of 1st versus later-borns in families with exactly 3 children (as of March 31st 2011); Table S2: Analyses of 1st versus later-borns Stratified by Mother's age. (DOCX) [file pone.0081070.s001.docx]

**Table S1: Analyses of 1st versus later-borns in families with exactly 3 children (as of March 31^st^ 2011).**

| Birth-order | Vaccinated Children | Events During Risk Period (Days  0-2) | Events During Control Period (Days 9-18) | Relative Incidence (95% CI) | Relative Incidence  Ratio (95% CI) | RIR  p-value |
| --- | --- | --- | --- | --- | --- | --- |
| 2 month Vaccination |  |  |  |  |  |  |
| 2^nd^ or higher | 41502 | 128 | 563 | 0.68 (0.56-0.83) | 1 (Ref) |  |
| 1^st^ | 3299 | 24 | 59 | 1.22 (0.76-1.96) | 1.79 (1.07-2.99) | 0.0259 |
| 4 month vaccination |  |  |  |  |  |  |
| 2^nd^ or higher | 39326 | 102 | 574 | 0.53 (0.43-0.66) | 1 (Ref) |  |
| 1^st^ | 3249 | 12 | 51 | 0.71(0.38-1.32) | 1.32 (0.68-2.57) | 0.4067 |
| 6 month vaccination |  |  |  |  |  |  |
| 2nd or higher | 37249 | 113 | 568 | 0.60 (0.49-0.73) | 1 (Ref) |  |
| 1st | 3149 | 19 | 73 | 0.78 (0.47-1.29) | 1.31 (0.76-2.25) | 0.3327 |
| 12 month vaccination |  |  |  |  |  |  |
| 2nd or higher | 34221 | 702 | 538 | 1.48 (1.07-2.06) | 1 (Ref) |  |
| 1st | 3019 | 89 | 60 | 1.30 (1.16-1.46) | 0.88 (0.62-1.24) | 0.4678 |

**Table S2: Analyses of 1st versus later-borns Stratified by Mother’s age**

|  |  | Two Month Vaccination | Four Month Vaccination | Six Month Vaccination | Twelve Month Vaccination |
| --- | --- | --- | --- | --- | --- |
| Mother Age Category | Birth-order | Relative Incidence  Ratio (95% CI) | Relative Incidence  Ratio (95% CI) | Relative Incidence  Ratio (95% CI) | Relative Incidence  Ratio (95% CI) |
| Overall | 2^nd^ or higher | 1 (Ref) | 1 (Ref) | 1 (Ref) | 1 (Ref) |
|  | 1^st^ | 1.37 (1.19, 1.57) | 1.70 (1.45, 1.99) | 1.27 (1.09, 1.48) | 1.11 (1.02, 1.21) |
| 16-20 | 2^nd^ or higher | 1 (Ref) | 1 (Ref) | 1 (Ref) | 1 (Ref) |
|  | 1^st^ | 0.94 (0.54,1.63) | 0.85 (0.46,1.57) | 1.09 (0.57,2.11) | 1.19 (0.73,1.94) |
| 21-25 | 2^nd^ or higher | 1 (Ref) | 1 (Ref) | 1 (Ref) | 1 (Ref) |
|  | 1^st^ | 1.39 (1.02,1.89) | 1.51 (1.07,2.13) | 1.27 (0.91,1.76) | 1.10 (0.91,1.34) |
| 26-30 | 2nd or higher | 1 (Ref) | 1 (Ref) | 1 (Ref) | 1 (Ref) |
|  | 1st | 1.37 (1.05,1.78) | 1.82 (1.36,2.43) | 1.16 (0.87,1.53) | 1.12 (0.97,1.30) |
| 31-35 | 2nd or higher | 1 (Ref) | 1 (Ref) | 1 (Ref) | 1 (Ref) |
|  | 1st | 1.33 (1.00,1.77) | 1.83 (1.33,2.50) | 1.39 (1.02,1.87) | 1.07 (0.92,1.25) |
| 36-40 | 2^nd^ or higher | 1 (Ref) | 1 (Ref) | 1 (Ref) | 1 (Ref) |
|  | 1^st^ | 1.39 (0.87,2.22) | 2.04 (1.24,3.37) | 1.15 (0.70,1.89) | 1.05 (0.81,1.35) |
| 41+ | 2^nd^ or higher | 1 (Ref) | 1 (Ref) | 1 (Ref) | 1 (Ref) |
|  | 1^st^ | 1.27 (0.40,4.05) | 1.05 (0.29,3.98) | 1.70 (0.58,4.99) | 0.97 (0.49,1.91) |
